# TUG1 Is a Regulator of AFP and Serves as Prognostic Marker in Non-Hepatitis B Non-Hepatitis C Hepatocellular Carcinoma †

**DOI:** 10.3390/cells9020262

**Published:** 2020-01-21

**Authors:** Yang-Hsiang Lin, Meng-Han Wu, Ya-Hui Huang, Chau-Ting Yeh, Kwang-Huei Lin

**Affiliations:** 1 Liver Research Center, Chang Gung Memorial Hospital, 15 Wen-hwa 1 Road, Linkou, Taoyuan 333, Taiwan; yhlin0621@cgmh.org.tw (Y.-H.L.); e1249060@gmail.com (Y.-H.H.); 2 Department of Biochemistry, College of Medicine, Chang Gung University, 259 Wen-hwa 1 Road, Taoyuan 333, Taiwan; snoopy740621@yahoo.com.tw; 3 Research Center for Chinese Herbal Medicine, College of Human Ecology, Chang Gung University of Science and Technology, Taoyuan 333, Taiwan; 4 Graduate Institute of Biomedical Sciences, College of Medicine, Chang Gung University, Taoyuan 333, Taiwan

**Keywords:** thyroid hormone, long non-coding RNA, alpha-fetoprotein, prognostic marker, overall survival

## Abstract

Thyroid hormone (T_3_) and its receptor (TR) are involved in cell metabolism and cancer progression. Hypothyroidism is associated with significantly elevated risk of hepatocellular carcinoma (HCC). Levels of the glycoprotein alpha-fetoprotein (AFP) are increased in the majority of patients with HCC and may be useful in diagnosis and follow-up. However, the relationship between T_3_/TR and AFP levels in HCC is currently unclear. The expression profiles of long non-coding RNAs (lncRNAs) were compared in microarrays of HepG2-TRα1 cells treated with/without T_3_ and HCC specimens. The effects of T_3_ on taurine upregulated gene 1 (*TUG1*) and *AFP* expression were validated using qRT-PCR. A correlation between TUG1 and AFP was confirmed via RNAi and clustered regularly interspaced short palindromic repeats (CRISPR) strategies. Finally, overall and recurrence-free survival rates were analyzed using the Kaplan–Meier method and confirmed in online datasets. T_3_/TR treatment reduced *TUG1* expression in vitro, resulting in the downregulation of *AFP* mRNA. Knockdown of *TUG1* suppressed cell cycle progression and soft agar colony formation and induced cellular senescence. Our data support the involvement of TUG1 in the T_3_/TR-mediated suppression of cell growth. *AFP* mRNA levels showed strong positive correlations with TUG1 and unfavorable prognosis in patients with non-hepatitis B/non-hepatitis C HCC (NBNC-HCC). T_3_/TR, TUG1, and AFP may potentially serve as effective prognostic markers for NBNC-HCC.

## 1. Introduction

Thyroid hormone (3,3′,5-triiodo-l-thyronine; T_3_) regulates cell homeostasis, growth, development, autophagy and metabolism [1] through binding to thyroid hormone receptors (TR) [2]. Human TRs are encoded by *TRα1* and *TRβ1* genes located on chromosomes 17 and 3, respectively [3]. Aberrant expression and/or mutation of *TRs* has been documented in pituitary tumors [4], hepatocellular carcinoma (HCC) [5] and thyroid cancer [6]. Hypothyroidism is associated with a significantly elevated risk for HCC, especially in hepatitis virus-negative subjects, non-drinkers, non-diabetics and non-smokers [7], along with non-alcoholic steatohepatitis (NASH) [8]. These findings indicate that T_3_/TR acts to suppress the development of liver cancer. However, the molecular mechanisms underlying the associations between T_3_/TR and HCC are yet to be elucidated.

HCC is one of the most common and aggressive human malignancies worldwide. The majority of patients with HCC have an established background of chronic liver disease and cirrhosis, with major etiological and risk factors including chronic infection with hepatitis B virus (HBV) and hepatitis C virus (HCV) [9]. The development of an HBV vaccine [10] and HBV screening for blood transfusion have effectively reduced the incidence of new HBV infections. Although most HCC cases are associated with viral infection, many patients are negative for both HBV and HCV (NBNC-HCC). Alcohol abuse, diabetes mellitus (DM), and obesity are contributory factors to alcohol-related liver disease (ALD) and NASH, which can trigger HCC development [11,12,13]. Aberrant expression of alpha-fetoprotein (*AFP*) is the most widely used biomarker for HCC surveillance [14]. *AFP* expression is regulated by genes encoding the proteins *p53* and *ZBTB20* and the small non-coding RNA *miR-122* [15,16]. Regulator-mediated AFP regulation is therefore currently a significant focus of cancer biology research.

Long non-coding RNAs (lncRNAs) are a class of non-protein coding transcripts longer than 200 nucleotides that regulate complex cellular functions, such as cell growth, metabolism, and metastasis [17]. A lncRNA, taurine upregulated gene 1 (*TUG1*), is involved in oncogenesis of various cancer types, including colorectal [18], pancreatic [19], and cervical cancer [20]. *TUG1* is highly expressed in tumors and shown to play an oncogenic role in HCC [21,22]. He and co-workers demonstrated that knockdown of *TUG1* and upregulation of *miR-142-3p* suppressed cell proliferation, migration, invasion and epithelial-mesenchymal transition (EMT) [23]. ZEB1 was identified as a target of *miR-142-3p*. Moreover, *miR-142-3p* was negatively regulated by TUG1. These findings support regulatory effects of the *TUG1*/*miR-142-3p*/*ZEB1* axis on HCC progression. Notably, TUG1 could regulate tumor progression by acting as a competing endogenous RNA (ceRNA) of miRNAs [24]. Lv et al. [25] demonstrated that TUG1 interactions with *miR-144* promote growth and migration of HCC cells through activation of the JAK2/STAT3 pathway. Yet another study reported that *TUG1* serves as competing endogenous RNA (ceRNA) by interacting with *miR-132* for binding the sonic hedgehog gene, leading to repression of tumorigenic activity [26]. Although TUG1 and AFP levels are reported to show a positive clinical correlation, the mechanisms linking T_3_/TR, TUG1 and AFP to HCC remain unclear. In the current study, we analyzed these associations in hepatoma cells overexpressing *TR* and samples from patients with HCC.

## 2. Materials and Methods

### 2.1. Cell Culture

HepG2, J7, Hep3B and SK-Hep1 cell lines were cultured in Dulbecco’s modified Eagle’s medium (DMEM) containing 10% (*v*/*v*) fetal bovine serum (FBS). HepG2 cells were transfected with pcDNA3 (designated HepG2-neo), pcDNA3-TRα1 (designated HepG2-TRα1) and pcDNA3-TRβ1 (designated HepG2-TRβ1), respectively, by the TurboFect transfection reagent (ThermoFisher Scientific, Kalamazoo, MI, USA) according to the manufacturer’s instructions. Stable cell lines, including HepG2-TRα1, HepG2-TRβ1 and HepG2-neo, were cultured in DMEM containing 10% (*v*/*v*) FBS and G418. Serum was depleted of T_3_ as described previously [27]. Cells were grown at 37 °C in a humidified atmosphere of 95% air and 5% CO_2_.

### 2.2. Human Hepatoma Specimens

Overall, 160 paired hepatoma specimens from Taiwan Liver Cancer Network (TLCN) were collected for study and subjected to qRT-PCR and western blot analyses. The protocol was approved by the Medical Ethics and Human Clinical Trial Committee at Chang-Gung Memorial Hospital.

### 2.3. Microarray Analysis

Sample preparation and microarray hybridization were performed using the Agilent SurePrint G3 Human V2 GE 8 × 60 K Microarray (Agilent Technologies, Santa Clara, CA, USA). T_3_/TR-regulated lncRNAs and mRNAs were identified by fold-change filtering (≥2.0 or ≤0.5), paired *t*-test (*p* < 0.05) and multiple hypothesis testing (FDR < 0.05).

### 2.4. Quantitative Reverse Transcription-PCR (qRT-PCR)

Total RNA was extracted from cells using TRIzol reagent (Life Technologies Inc., Carlsbad, CA, USA) and cDNA was synthesized using ToolScript MMLV RT kit (BIOTOOLS CO., LTD. Taiwan). qRT-PCR was performed in 15 µL reaction mixtures containing forward and reverse primers and 1X SYBR Green mix (Applied Biosystems, Carlsbad, CA, USA). The amplification protocol consisted of an initial denaturation at 95 °C for 10 min, 40 cycles of denaturation at 95 °C for 15 s, and annealing and extension at 60 °C for 1 min, followed by a dissociation step. All reactions were performed in an ABI Prism 7500 Fast Real-Time PCR system (Life Technologies). The primer sequences for TUG1 were 5′-CTCTCTTTACTGAGGGTGCTTTAGCT-3′ (forward) and 5′-TCTCTCCATATTTTGGCTCTGCTT-3′ (reverse); the sequences for 18S rRNA were 5′-CGAGCCGCCTGGATACC-3′ (forward) and 5′-CCTCAGTTCCGAAAACCAACAA-3′ (reverse); the sequences for GAPDH were 5′-AATCCCATCACCATCTTCCA-3′ (forward) and 5′-TGGACTCCACGACGTACTCA-3′ (reverse); and the sequences for AFP were 5′-CCCGAACTTTCCAAGCCATA-3′ (forward) and 5′-TACATGGGCCACATCCAGG-3′ (reverse).

### 2.5. Immunoblot Analysis

Immunoblot analysis was performed as described previously [28], using antibodies specific for AFP, PCNA, cyclin E, Lamin A/C (Santa Cruz Biotechnology Inc., Santa Cruz, CA, USA), active caspase-3 (Abcam, Cambridge, MA, USA), Cleavaged PARP (BD Biosciences, San Jose, CA, USA), EZH2, SUZ12, H3K27me3 and GAPDH (Merck Millipore, Billerica, MA, USA).

### 2.6. Establishment of TUG1 Knockdown and TUG1-Activating Cell Lines

Clones of shRNA targeting the *TUG1* gene were purchased from the National RNA Interference Core Facility (Institute of Molecular Biology, Academia Sinica, Taipei, Taiwan) and listed in Supplementary Table S1. ShRNA lentivirus was generated, and cells with stable knockdown selected in medium containing puromycin. It is very difficult to construct full-length *TUG1* sequences (about 7.2 kb) to an expression vector. Alternatively, the CRISPR-Cas9 activation system was used to generate *TUG1* overexpression cells. Three sgRNAs targeting the TUG1 promoter sequences were designed using a free online sgRNA design tool. The gRNA cloning vector (Addgene #41824) was linearized by Afl II and incorporate the interested DNA fragment (sgRNA) into the linearized vector using the Gibson assembly. Furthermore, the sequence of gRNAs (TUG1-sgRNA#1, TUG1-sgRNA#2 and TUG1-sgRNA#3) was confirmed by sequencing. Cells were co-transfected with various plasmids, including dCas9-VP64 (Addgene plasmid #61422), U6-sgRNA (empty vector), U6-TUG1-sgRNA#1, U6-TUG1-sgRNA#2 and U6-TUG1-sgRNA#3 using TurboFect transfection reagents (ThermoFisher Scientific) according to the manufacturer’s instructions.

### 2.7. Detection of Cell Cycle Progression

Initially, cells were starved in serum-free medium for 24 h. Subsequently, cells were treated with or without T_3_ 10 nM. After 24 h of treatment, cells were washed twice with cold PBS and harvested with trypsin. Cells were fixed with 70% ethanol for 1 h at −20 °C and incubated in 0.5% Triton X-100/PBS containing 0.05% DNase-free RNase for 1 h at 37 °C. Then, cells were stained with propidium iodide (Sigma-Aldrich, St Louis, MO, USA), and cell cycle progression was determined using flow cytometry.

### 2.8. Cell Proliferation Assay

Stable cells (4 × 10^3^/well) were grown on a 6-well plate. After 48 h of T_3_ treatment, cell growth rates were determined with trypan blue exclusion and quantitated using the LUNA*™* Automated Cell Counter.

### 2.9. Soft Agar Assay

Stable cells were suspended in 0.33% agar in DMEM containing 10% FBS. After three weeks, colonies were stained with crystal violet. Images were obtained under a microscope, and colony numbers counted with Image J (version 1.48, National Institutes of Health, Bethesda, Maryland, USA).

### 2.10. TUNEL Assay

Knockdown of *TUG1* in Huh7 and SK-Hep1 cells were seeded to a 6-well plate with a coverslip in the bottom. After 72 h, the apoptosis status was detected by the TUNEL assay as described previously [29].

### 2.11. Public Datasets

HCC datasets used in this study were GSE101679 [22], GSE14520 [30], GSE14323 [31], GSE62232) [32] and GSE45436.

### 2.12. Statistical Analysis

Results are presented as mean ± SD of three independent experiments. The Mann-Whitney *U*-test was used for comparisons of two groups and one-way ANOVA followed by Tukey post-hoc test for more than two groups. Survival outcomes (OS and RFS with death as an event) were generated using the Kaplan–Meier method and compared using the log-rank test. All statistical analyses were performed using SPSS version 20 software (SPSS Inc., Chicago, IL, USA), with *p* values < 0.05 considered statistically significant.

## 3. Results

### 3.1. TUG1 is Significantly Downregulated by T_3_/TR In Vitro and Regulates Cell Cycle Progression, Cellular Senescence and Soft Agar Colony Formation

**Figure 1:**
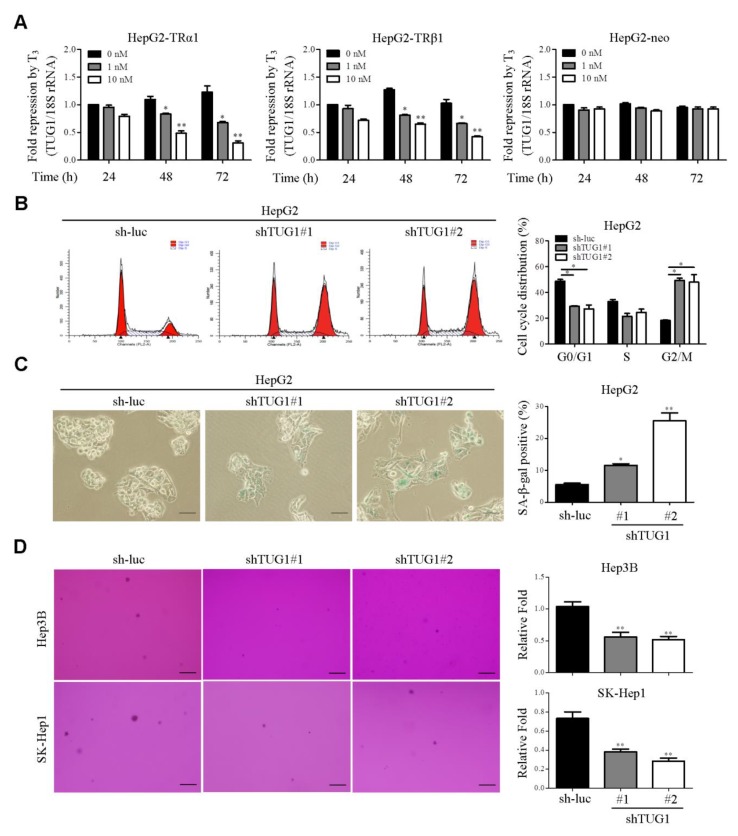
TUG1 is downregulated by T_3_/TR and regulates cell cycle progression, cellular senescence and colony formation. (**A**) HepG2-TRα1, HepG2-TRβ1 and HepG2-neo cells were treated with/without T_3_ for 24–72 h. TUG1 levels were measured using qRT-PCR and normalized to that of 18S rRNA. (**B**) Cell cycle progression was measured in control (sh-luc) and TUG1-depleted cells (shTUG1#1 and #2). (**C**) Cellular senescence was analyzed in stable *TUG1* knockdown cells. SA-β-gal positive cell numbers were calculated as shown. (**D**) Effects of TUG1 on soft agar colony formation. Scale bar, 100 μm. *, *p* < 0.05; **, *p* < 0.01.

**Figure 2:**
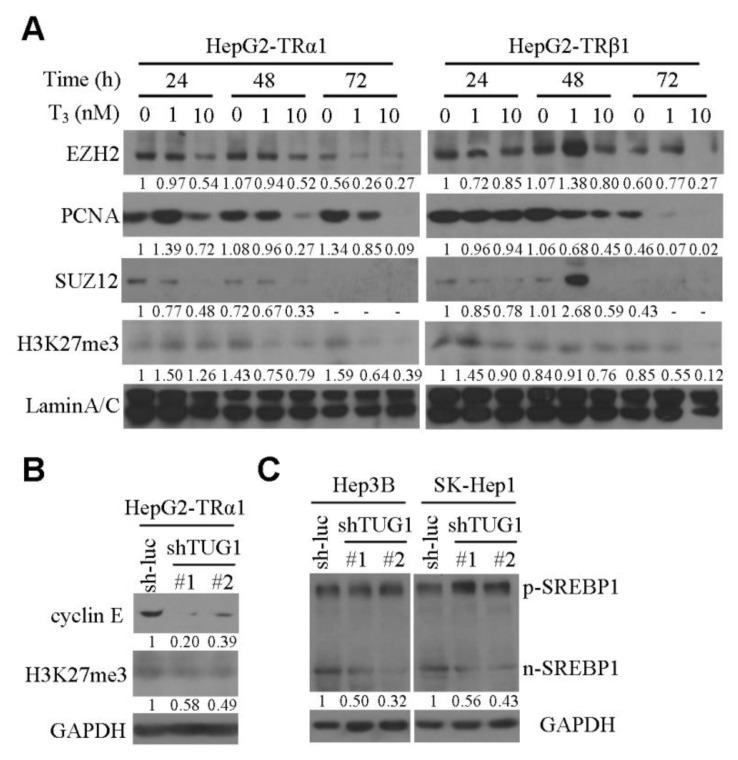
*TUG1* regulates the epigenetic markers cyclin E and SREBP1. (**A**) HepG2-TRα1 and HepG2-TRβ1 cells were treated with/without T_3_ for 24–72 h, and EZH2, SUZ12, H3K27me3 and PCNA levels determined via western blot using lamin A/C as the loading control. (**B**) Expression of cyclin E and *H3K27me3* determined in TUG1-depleted cells using GAPDH as the loading control. (**C**) Expression of *SREBP1* determined in TUG1-depleted Hep3B and SK-Hep1 cells using GAPDH as the loading control.

Microarray analysis of HepG2-TRα1 cells treated or not with 20 nM T_3_ for 24 and 48 h led to the identification of several T_3_/TR-regulated lncRNAs. We focused on the lncRNAs upregulated ≥ 2-fold (*p* < 0.05) and downregulated ≥ 2.0-fold (*p* < 0.05) by T_3_/TR (Supplementary Figure S1A). Among the candidate lncRNAs simultaneously downregulated by T_3_/TR and upregulated in HCC (GSE101679) [22], TUG1 was selected for further study (Supplementary Figure S1A). qRT-PCR conducted to verify gene expression results obtained from microarray analysis confirmed significant downregulation of *TUG1* by T_3_/TR in HepG2-TRα1 and HepG2-TRβ1 cells (Figure 1A). In contrast, *TUG1* expression was only marginally downregulated by T_3_ in HepG2-neo cells (Figure 1A). To elucidate the mechanisms underlying T_3_-mediated suppression of TUG1, RNA stability was investigated by suppressing new RNA synthesis in HepG2-TR with actinomycin D (ActD). Following ActD treatment, the half-life of TUG1 RNA in HepG2-TR cells was similar in the presence and absence of T_3_ (Supplementary Figure S1B), indicating that repression of TUG1 RNA synthesis by T_3_ is not mediated by variations in RNA stability. To further clarify the regulatory effect of T_3_ on *TUG1* expression, cycloheximide (CHX), a protein synthesis inhibitor, was introduced. The effect of T_3_ on *TUG1* expression in the presence and absence of CHX was determined in HepG2-TRα1 cells. Blockage of protein repression with CHX partially abolished transcriptional repression of TUG1 by T_3_ (Supplementary Figure S1C), suggesting that the regulatory effect of T_3_ on TUG1 is indirect and mediated by another transcription factor. TUG1 is reported to be positively regulated by SP1 [21], which is significantly negatively regulated by T_3_/TR [27]. To further address whether TUG1 is regulated by SP1, *TUG1* in SP1-overexpressing cells was assessed using qRT-PCR. TUG1 upregulation by SP1 was observed in hepatoma cells (Supplementary Figure S1D, left panel). Moreover, *TUG1* expression was clearly suppressed in T_3_-treated cells relative to those without T_3_. Interestingly, this effect was partially restored by *SP1* overexpression in the presence of T_3_ (Supplementary Figure S1D, right panel). Based on these findings, we propose that TUG1 is partially downregulated by T_3_ through suppression of SP1. The diagnostic ability of TUG1 was evaluated based on the receiver operating characteristic (ROC) area under curve (AUC), calculated as 0.809 (Supplementary Figure S1E). Knockdown of *TUG1* suppressed soft agar colony formation and induced cell cycle arrest at the G2/M phase, along with cellular senescence (Figure 1B–D). The effect of TUG1 on apoptosis was additionally assessed via the TUNEL assay. The results indicate that knockdown of TUG1 accelerates apoptosis (Supplementary Figure S2A). Expression levels of active caspase-3 and cleaved *PARP*, determined via western blot, were increased in the presence of TUG1-specific shRNA (Supplementary Figure S2B). Previous studies have shown that *TUG1* regulates gene expression through interactions with polycomb repressive complex (PCR) 2 [21]. Accordingly, the effects of T_3_/TR on PRC2 core components were determined in HepG2-TR cell lines. EZH2, SUZ12 and trimethylation at lysine 27 of histone 3 (H3K27me3) were downregulated following T_3_ treatment of HepG2-TR cells (Figure 2A). Moreover, proliferating cell nuclear antigen (PCNA), a cell cycle marker, was remarkably suppressed by T_3_/TR (Figure 2A). Cyclin E and H3K27me3 levels were reduced in TUG1-depleted HepG2-TRα1 cells (Figure 2B). The collective findings suggest that cyclin E and epigenetic markers are downregulated by T_3_/TR, which may be mediated through repression of TUG1. Interestingly, the active form of sterol regulator element binding protein (SREBP)-1 involved in the biogenesis of cholesterol, fatty acids and triglycerides [33] was suppressed in TUG1-depleted cell lines (Figure 2C).

### 3.2. AFP is Significantly Downregulated by the T_3_/TR/TUG1 Axis

**Figure 3:**
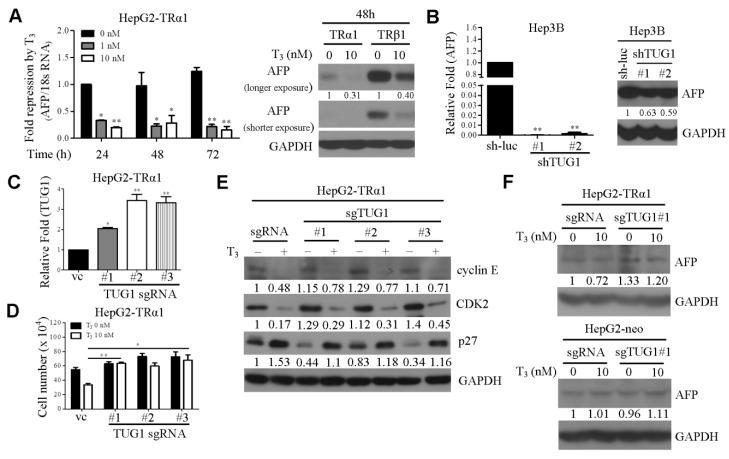
AFP is downregulated by T_3_/TR through suppression of TUG1. (**A**) HepG2-TRα1 cells were treated with/without T_3_, and *AFP* mRNA and protein levels measured via qRT-PCR and western blot analysis using 18S rRNA and GAPDH as the loading controls, respectively. (**B**) qRT-PCR and western blot analysis of *AFP* expression in *TUG1* knockdown cell lines using GAPDH as the loading control. (**C**) qRT-PCR analysis of *TUG1* expression in TUG1-activated cell lines using 18S rRNA as the loading control. (**D**) Cell growth was determined in the indicated cells treated with/without T_3_. (**E**) HepG2-TRα1 *TUG1*-overexpressing cells were treated with T_3_, and levels of the cell cycle-related proteins (cyclin E, CDK2 and p27) measured via western blot with GAPDH as the loading control. (**F**) Western blot analysis of *AFP* expression in HepG2-TRα1 or HepG2-neo *TUG1*-overexpressing cells treated with T_3_ (10 nM) using GAPDH as the loading control. *, *p* < 0.05; **, *p* < 0.01.

As mentioned above, AFP is well known clinical marker in the diagnosis and treatment of liver cancer. Multiple lines of evidence suggest that AFP acts as signaling regulator to modulate cell proliferation, cell cycle, apoptosis and migration [16,34]. Recent experiments by Wang et al. [35] demonstrated that AFP suppresses autophagy and apoptosis and promotes cell growth, migration and invasion though interactions with PTEN. To test the hypothesis that AFP participates in T_3_/TR-mediated functions, *AFP* expression following T_3_ treatment was initially determined. As expected, *AFP* mRNA and protein levels were dramatically reduced by T_3_/TR (Figure 3A). We had previously shown a positive correlation between *TUG1* and *AFP* expression [22]. To further establish whether *TUG1* mediates *AFP* expression, TUG1 depletion was achieved in Hep3B cells and *AFP* mRNA levels assayed via qRT-PCR. Notably, the knockdown of *TUG1* led to significant reduction of *AFP* mRNA and protein levels (Figure 3B). These findings indicate that AFP is downregulated by T_3_/TR and decreased in TUG1-depleted cell lines in vitro.

### 3.3. TUG1 is Involved in T_3_-Mediated Functions In Vitro

Accumulating evidence has shown that T_3_ suppresses cell growth, both in vitro and in vivo [27]. To determine the involvement of TUG1 in T_3_/TR-regulated cell proliferation ability, its overexpression in HepG2-TRα1 was established using CRISPR-mediated transcriptional activation (CRISPRa). HepG2-TRα1 cells were co-transfected with individual sgRNA (TUG1 sgRNA#1, #2 and #3) constructs targeting TUG1 promoter regions and dCas9-VP64 plasmid, respectively, and qRT-PCR performed to determine TUG1 activation patterns (Figure 3C). Cell proliferation in the presence and absence of T_3_ was additionally examined in these cell lines. T_3_ clearly inhibited growth of HepG2-TRα1-vc/dCas9-VP64 cells relative to control cells without T_3_. Interestingly, this effect was partially promoted by TUG1 activation (sgRNA#1, #2 and #3) in the presence of T_3_ (Figure 3D). In addition, protein levels of cyclin E and CDK2 were clearly decreased upon T_3_ treatment (Figure 3E). p27 was upregulated by T_3_/TR in the control group. These effects were partially rescued by TUG1 activation in the presence of T_3_ (Figure 3E). To ascertain whether AFP protein is regulated by the T_3_/TUG1 axis, total proteins in TUG1-activating and control cell lines were extracted and subjected to western blot analysis. AFP downregulation by T_3_/TR was partially blocked upon overexpression of *TUG1* in HepG2-TRα1 cells (Figure 3F). Analysis of the effects of T_3_/TR on apoptosis in TUG1-activated cells clearly revealed the suppression of active caspase-3 in T_3_-treated (Supplementary Figure S2C, Lane 1 vs. Lane 2) as well as TUG1-activated cells (Supplementary Figure S2C, Lane 1 vs. Lane 3). These findings collectively support a role of TUG1 as a tumor promoter and its involvement in T_3_-mediated cell growth in hepatoma.

### 3.4. Clinical Correlation between TUG1 and AFP

**Figure 4:**
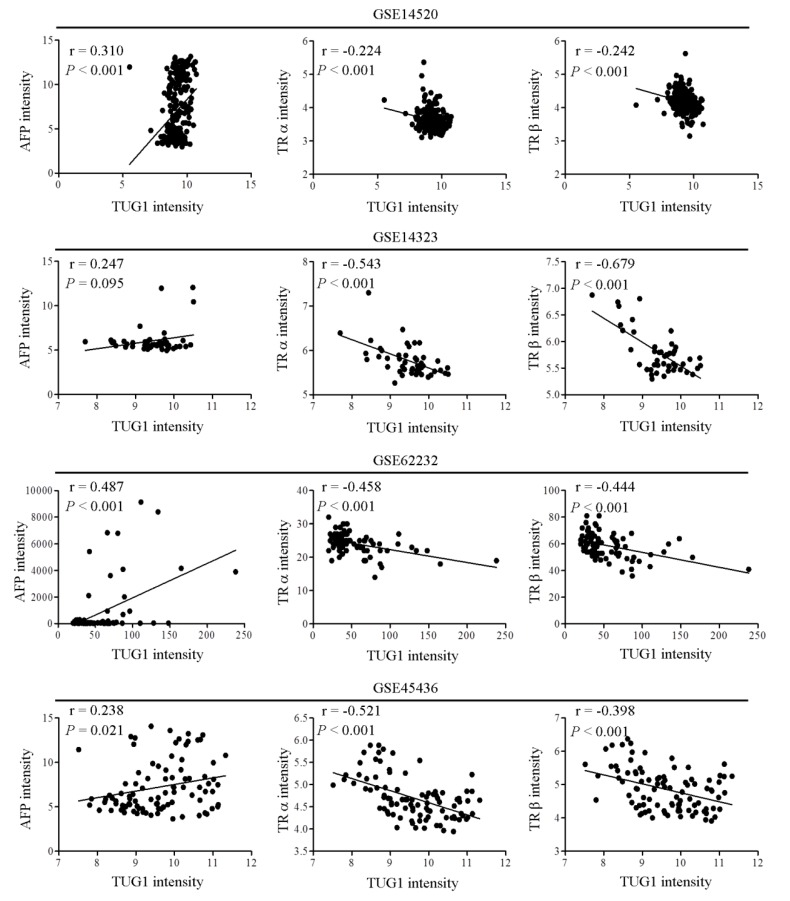
TUG1 is positively and negatively associated with AFP and TR, respectively. Correlations among TUG1, *AFP* mRNA and TR levels in HCC tissue samples. Pairwise correlations were determined using Pearson correlation analysis.

To further confirm whether *AFP* expression was associated with HCC progression in our cohort, *AFP* mRNA levels were measured via qRT-PCR in HCC specimens and surrounding non-tumor tissue. Our results disclosed higher *AFP* mRNA expression in HCC than non-tumor tissue samples (Supplementary Figure S3A). Kaplan–Meier analysis showed association of high *AFP* expression with poor overall survival (OS) and poor recurrence-free survival (RFS) in patients with HCC (Supplementary Figure S3B). Furthermore, we observed significant positive correlations of AFP with gender and cirrhosis (Table 1). Pearson correlation analysis revealed that *TUG1* and *AFP* mRNA levels were significantly positively correlated (Supplementary Figure S3C), which was confirmed by the analysis of four public datasets (GSE14520, GSE14323, GSE62232, and GSE45436) of HCC samples (Figure 4). Moreover, TUG1 was significantly negatively correlated with TR in HCC tumors (Figure 4). 

### 3.5. Combined Expression of TUG1 and AFP is a Stronger Predictor of OS and RFS in Patients with NBNC-HCC

**Figure 5:**
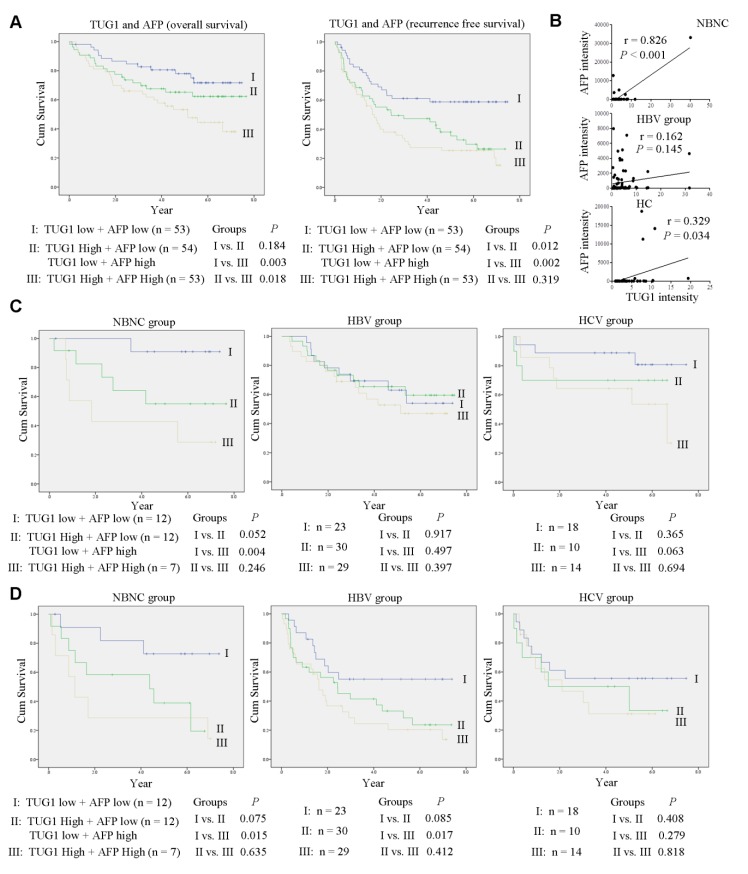
The TUG1/AFP axis is correlated with poor prognosis in patients with NBNC-HCC. (**A**) A total of 160 HCC patients were categorized into three groups based on *TUG1* and *AFP* mRNA levels. High levels of both TUG1 and AFP were associated with significantly poorer OS and RFS, as determined using the log-rank test. (**B**) Correlations of *TUG1* and *AFP* mRNA levels in patients with NBNC-HCC, HBV-HCC and HCV-HCC using Pearson correlation analysis. Kaplan-Meier analysis of OS (**C**) and RFS (**D**) in patients with NBNC-HCC, HBV-HCC and HCV-HCC at high or low risk of survival. *p* values were determined using the log-rank test.

In view of the significant positive correlation between *TUG1* and *AFP* mRNA levels in HCC tumors, we evaluated their combined effects on outcomes in 160 patients. Patients were categorized into three groups based on median *TUG1* and *AFP* mRNA levels in HCC tumors, with Group I consisting of 53 patients with low *TUG1* and low *AFP*, Group II including 54 patients with low *TUG1* and high *AFP* or high *TUG1* and low *AFP*, and Group III comprising 53 patients with high *TUG1* and high *AFP*. Notably, patients in Group III showed significantly poorer OS and RFS relative to those in Groups I and II (Figure 5A).

Patients were additionally classified into four etiologic groups (31 with NBNC, 82 positive for HBV alone, 42 positive for HCV alone and 5 positive for HBV and HCV). The strongest positive correlation between *TUG1* and *AFP* expression was evident in the NBNC group (Figure 5B). Analysis of the combined effects of etiology, *TUG1* and *AFP* expression revealed that OS and RFS were significantly poorer in patients with NBNC along with Group III showing higher expression of both *TUG1* and *AFP*, compared to the other groups (Figure 5C,D). These findings support the utility of *TUG1* and *AFP* as potential prognostic biomarkers for NBNC-HCC.

## 4. Discussion

The present study showed that T_3_/TR downregulates *TUG1* and *AFP* mRNA and protein levels, with knockdown of *TUG1* leading to dramatic reduction of *AFP* expression in Hep3B cells. Additionally, we observed a positive correlation between *TUG1* and *AFP* expression in HCC samples and association of high expression of *AFP* with significantly poorer OS and RFS. Finally, a combination of high *TUG1* and high *AFP* was particularly predictive of poorer OS and RFS in patients with NBNC-HCC. Our data suggest that TUG1 is involved in T_3_/TR-induced suppression of cell proliferation and may mediate this activity by modulation of cell cycle-related genes and PRC2 activity.

Gene dysregulation is a hallmark of cancer [36]. Experiments from the current study showed that TUG1 is positively regulated by SP1, consistent with previous reports [21]. Earlier, our group demonstrated that *SP1* is downregulated by T_3_ in *TR*-overexpressing cells and parental cells expressing endogenous TR [27,37]. Our collective findings provide evidence that TUG1 is partially downregulated by T_3_/TR though the suppression of SP1. The group of Li [38] demonstrated that ectopic expression of Forkhead Box M1 (*FOXM1*) led to upregulation of *TUG1* in osteosarcoma cell lines while a mutant form of *FOXM1* did not influence *TUG1* levels. *FOXM1* was additionally downregulated by T_3_/TR (data not shown). These associations provide evidence of the involvement of other regulatory mechanisms in FOXM1-mediated modulation of *TUG1* expression.

We previously showed that several protein-coding genes, including those encoding endoglin [39], pituitary tumor-transforming 1 (PTTG1) [27], Dickkopf 4 (DKK4) [40], ubiquitin-like with PHD and ring finger domains 1 (UHRF1) [37], and death-associated protein kinase 2 (DAPK2) [1], as well as non-coding genes, such as microRNA-214 (*miR-214*) [41] and BC200 [42], are regulated by T_3_/TR in HepG2-TR cell lines. The finding that TUG1 is downregulated by T_3_/TR, together with previously demonstrated oncogenic activity of TUG1 in HCC suggests that T_3_/TR suppresses cell growth by regulating protein-encoding genes and non-coding RNAs. These results indicate that TR performs a tumor suppressor role in HCC through reducing cell proliferation. However, the precise role of T_3_/TR in cancer is controversial. Although the effects of TUG1 on apoptosis were similar to those reported previously [21], earlier experiments by our group showed that *TR*-overexpressing hepatoma cells treated with T_3_ were apoptosis-resistant [43,44], suggesting that the effect of T_3_/TR on apoptosis is not mediated by TUG1.

PRC2 is involved in regulation of cell growth, cell cycle progression, cell senescence and metastasis [45,46]. TUG1 is a well-known EZH2-interacting lncRNA that regulates the oncogenic pathway to promote tumor progression [21]. An earlier study by Zhang et al. [47] showed that knockdown of *TUG1* suppresses gastric cancer growth. Furthermore, TUG1 interacted with PRC2 to induce epigenetic regulation of p15, p16, p21, p21, and p57. Here, we demonstrated that TUG1 is involved in T_3_/TR-mediated reduction of cell growth through regulation of cyclin E, CDK2, and p27. Moreover, levels of components (EZH2 and SUZ12) of PRC2 and H3K27me3 were reduced following T_3_ treatment of HepG2-TR cells. On the other hand, previous studies have documented that cyclin E/CDK2 phosphorylates EZH2 at T416 (pT416-EZH2) [48]. Phosphorylation of EZH2 at T416 promotes its ability to induce triple-negative breast cancer cell migration, invasion and tumor formation in vitro and in vivo. Based on these findings, it is suggested that cyclin E/CDK2 is an upstream regulator of EZH2. Previously, we reported significant downregulation of cyclin E and CDK2 by T_3_/TR [49], raising the possibility that T_3_/TR-mediated PRC2 activity is regulated via modulation of TUG1 or cyclin E/CDK2.

The regulation of *AFP* gene expression is a complex process involving several transcriptional activators and repressors that bind the AFP promoter region [15,16,50,51]. The precise mechanism by which TUG1 regulates *AFP* expression is currently unclear. Experiments from the present study demonstrated that both T_3_/TR and decreased *TUG1* expression were associated with reduced levels of *AFP* mRNA. Moreover, activation of *TUG1* could rescue *AFP* downregulated by T_3_ in HepG2-TRα1 but not HepG2-neo cells. These findings suggest that the *AFP* gene is negatively regulated by T_3_/TR through repression of TUG1. Notably, TUG1 is proposed to act as a ceRNA of miRNAs, supporting AFP regulation by the TUG1/miRNA axis as another potential mechanism.

Traditional AFP is widely used in the diagnosis, prognosis, and surveillance of HCC. However, the utility of AFP as a biomarker for HCC is a controversial issue at present. Owing to low specificity and sensitivity, AFP is inadequate for effective HCC surveillance [52,53]. For instance, higher expression may be detected in patients with cirrhosis or cholangiocarcinoma (low specificity) or normal levels in patients with early-stage HCC (low sensitivity). In a previous study, co-treatment with DKK1 and AFP improved the sensitivity and specificity of HCC detection, compared to individual tests [54]. Tomimaru and co-workers demonstrated that a combination of *AFP* and *miR-21* improved accuracy of detection of chronic hepatitis and HCC [55]. Furthermore, several biomarkers, including *Lens culinaris* agglutinin-reactive fraction of AFP, des-γ-carboxyprothrombin, glypican-3, and osteopontin, have been identified as a substitute or complement for AFP in the diagnosis and prognosis of HCC [34,56,57]. These findings suggest that combined testing with AFP and other biomarkers could help increase accuracy of diagnosis and prognosis of HCC. Data from the current study showed that AFP alone served as an effective single prognostic marker. Furthermore, the combination of AFP and TUG1 had good prognostic performance, compared to all other groups.

The incidence of HBV and HCV infections as a cause of HCC has been decreasing due to the successful development of vaccines and treatments with nucleoside/nucleotide analogs and interferon. The resulting relative increase in incidence of NBNC-HCC has led to the requirement for identifying novel biomarkers for HCC and reducing the unfavorable outcome rate. This study showed a strong positive correlation of AFP with TUG1. Moreover, combined expression of *TUG1* and *AFP* was a stronger predictor of OS and RFS in patients with NBNC-HCC than those with HBV-HCC and HCV-HCC. Based on the results, we propose that reduced TUG1 function is an important contributor to regulation of *AFP* expression in hepatoma cell lines and T_3_/TR, TUG1 and AFP may serve as potential prognostic biomarkers for NBNC-HCC. However, studies on larger patient cohorts are recommended for clinical validation.

Biomarkers should be non-invasive and easily accessible. Recently, circulating RNA levels in serum and plasma have been widely investigated as non-invasive biomarkers [58]. In a study by Li and co-workers [59], several lncRNAs, including TUG1, were detected in plasma of patients. A comparison of our findings to those of the earlier report revealed that plasma levels of TUG1 and AFP were correlated, especially in patients with NBNC-HCC.

In conclusion, T_3_/TR regulates expression of *TUG1* and *AFP* in a hepatoma cell line, which are significantly positively correlated. These biomarkers are associated with both OS and RFS, particularly in the NBNC-HCC subgroup of patients. Our collective results strongly support the utility of T_3_/TR, TUG1, and AFP as prognostic markers in patients with NBNC-HCC.

## Figures and Tables

**Table 1:** Clinicopathological correlations of AFP in HCC specimens.

Parameters	n = 160	AFP Mean *^a^* ± SE	*p ^b^*
Age (years)			
<65	101	1006 ± 366.9	0.8646
≥65	59	1108 ± 456.2	
Gender			
Male	80	1167 ± 514.8	0.0106
Female	80	920.5 ± 250	
Cirrhosis			
No	97	573.8 ± 171.9	0.0407
Yes	63	1767 ± 667.9	
Viral status			
NBNC	31	1852 ± 1134	
HBV	82	772.6 ± 181.3	0.6999
HCV	42	1100 ± 603	
Tumor type			
Solitary	127	848 ± 298.9	0.1794
Multiple	33	1794 ± 765.5	
Tumor size			
<5 cm	93	917.9 ± 296.8	0.6052
≥5 cm	67	1218 ± 545.3	
Vascular invasion			
No	81	555.1 ± 214.5	0.083
Yes	79	1545 ± 530.6	
Pathological stage			
I	76	640.3 ± 235.2	0.4044
II	52	1448 ± 728.5	
III	32	1345 ± 572.9	
Grading			
1	4	688.3 ± 687.1	0.0648
2	112	699.2 ± 227.2	
3	44	1953 ± 852.4	

^*a*^: Mean of *AFP* expression (T/N ratio). ^*b*^: Mann-Whitney *U* test (for two groups) or Kruskal Wallis test (for more than two groups).

## Supplementary Materials

The following are available online at https://www.mdpi.com/2073-4409/9/2/262/s1, Supplementary Figure S1. TUG1 is indirectly regulated by T_3_/TR, Supplementary Figure S2. Effect of TUG1 on apoptosis determined in hepatoma cell lines, Supplementary Figure S3. Overall and recurrence-free survival rates of HCC patients in relation to *AFP* expression, Table S1. List of TUG1 shRNA and sgRNA sequences used in this study.

## Abbreviations

T_3_	thyroid hormone
TR	thyroid hormone receptor
HCC	hepatocellular carcinoma
AFP	alpha-fetoprotein
lncRNA	long non-coding RNA
TUG1	taurine upregulated gene 1
qRT-PCR	quantitative reverse transcription-PCR
OS	overall survival
RFS	recurrence-free survival
