# Supplementary material for: TUG1 Is a Regulator of AFP and Serves as Prognostic Marker in Non-Hepatitis B Non-Hepatitis C Hepatocellular Carcinoma
Source: Cells. 2020 Jan 21;9(2):262. doi: 10.3390/cells9020262 (PMC7072672; doi:10.3390/cells9020262)
Supplement: Supplementary file 1 [file cells-09-00262-s001.pdf]

Table S1. List of the TUG1 shRNA and sgRNA sequences used in this study.

Name (Clone ID)	target sequence
shTUG1#1 (TRCN0000139193)	CTGTTGACCTTGCTGTGAGAA
shTUG1#1 (TRCN0000145288)	GCTCCATCCAAAGTGAATTAT
sgTUG1#1	GATCCGGGTAGTGCCCGGTCAGG
sgTUG1#2	CACTATCGGAGACAAAGCGGTGG
sgTUG1#3	GGACACGCAGCCCGCCAATCAGG

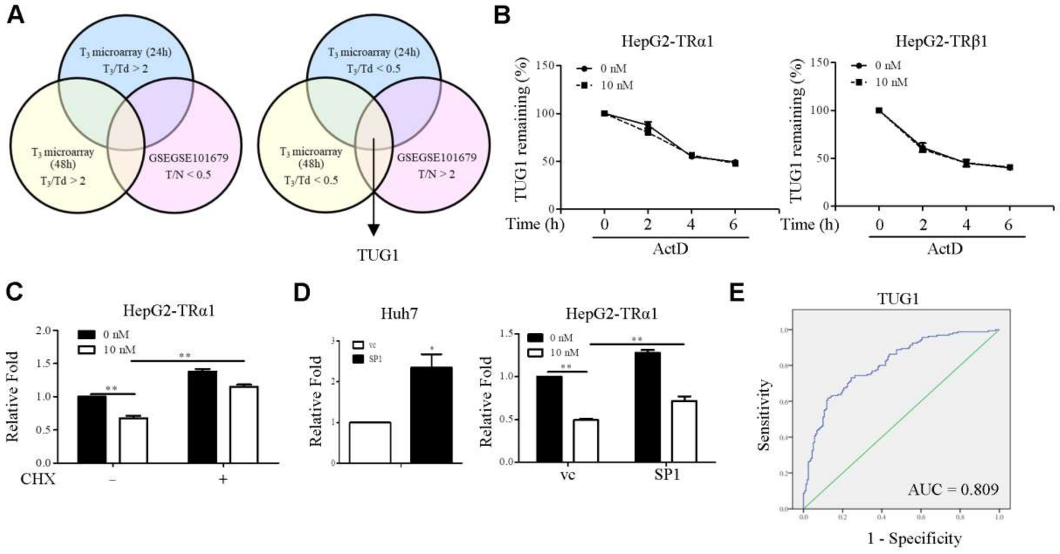

Supplementary Figure 1

Supplementary Figure 1. TUG1 is indirectly regulated by T_3/TR.

(A) Venn diagram for overlapping lncRNAs in the three microarray analysis. (B) T_3 does not affect the stability of TUG1 RNA. After T_3 treatment for 24 h, HepG2-TR cells were treated with actinomycin D (ActD, 2 μg/ml) for the indicated times. Total RNA was extracted and subjected to qRT-PCR analysis. (C) HepG2-TRα1 cells were co-treated with/without CHX and T_3, and TUG1 RNA levels measured using qRT-PCR, with 18S rRNA used as a loading control. (D) TUG1 RNA levels were meaured in SP1-overexpressing Huh7 and HepG2-TRα1 cell lines. 18S rRNA was used as a loading control. (E) ROC analysis of HCC-related lncRNA biomarker. AUC, area under the curve.

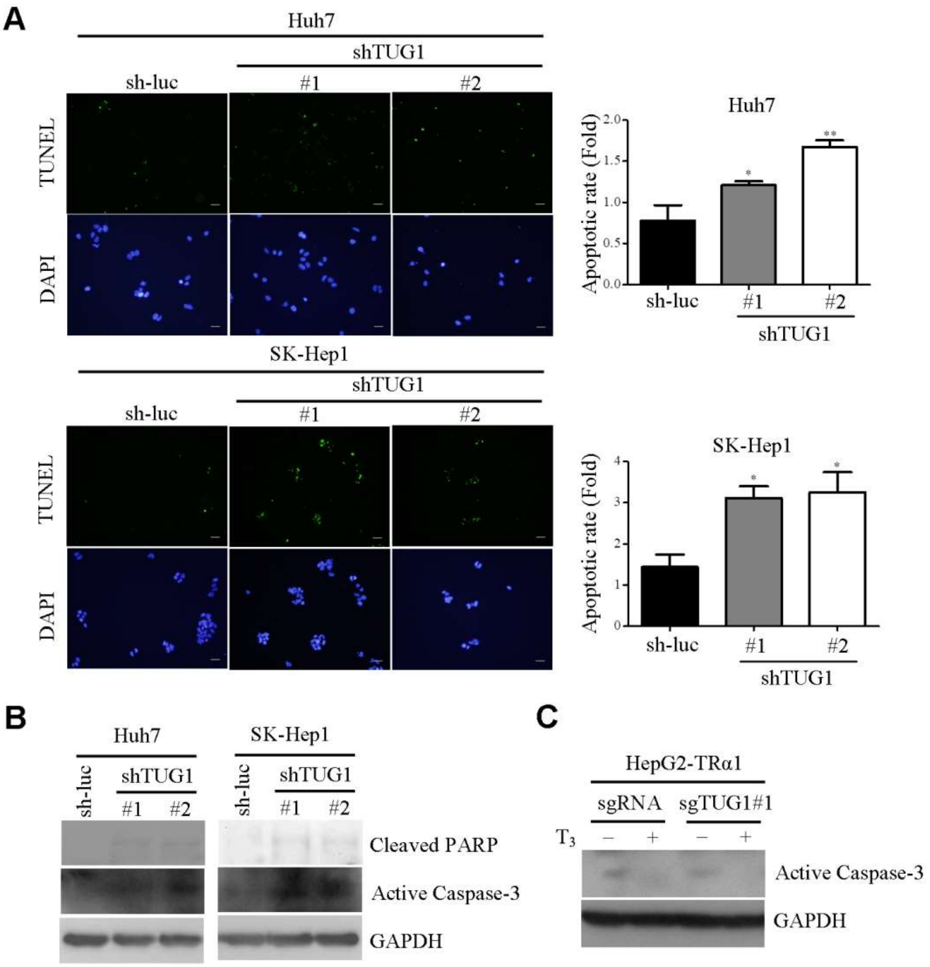

Supplementary Figure 2. Effect of TUG1 on apoptosis determined in hepatoma cell lines.

(A) TUNEL staining showed knockdown of TUG1 in Huh7 and Sk-Hep1 cells accelerated apoptosis. (B) Expression levels of caspase-3 and PARP were determined in TUG1-depleted cells. GAPDH was used as a loading control. (C) Western blot analysis of active caspase-3 expression in TUG1-overexpressing cells was treated with T_3 (10 nM). GAPDH was used as the loading control.

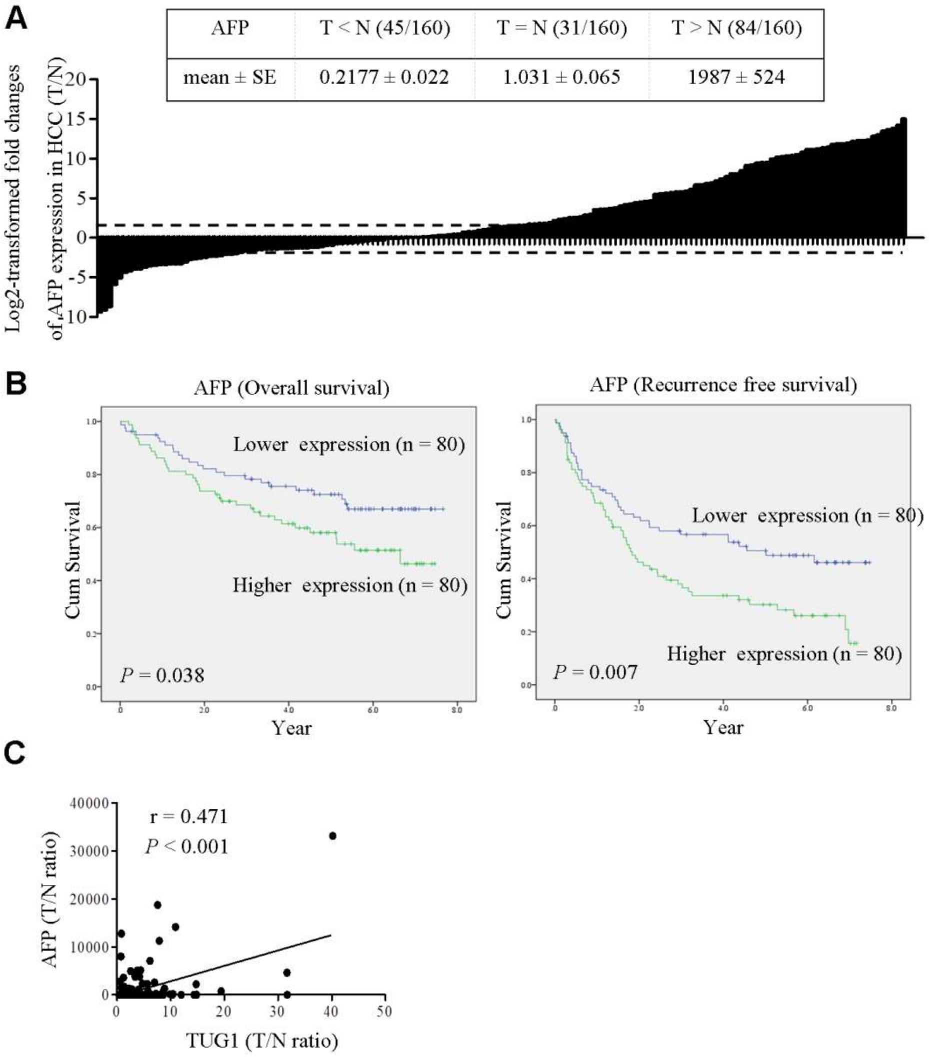

Supplementary Figure 3. Overall and recurrence-free survival rates of HCC patients in relation to AFP expression.

(A) AFP mRNA levels were analyzed in HCC specimens via qRT-PCR with 18S rRNA as the loading control. Values are expressed as log 2-transformed relative fold decrease or increase in mRNA expression, relative to that in adjacent nontumorous tissues after normalization to the housekeeping gene. A positive log 2-transformed fold change indicates higher expression in tumor specimens whereas a negative value signifies relatively decreased expression. (B) Kaplan-Meier analysis of overall survival (OS) and recurrence-free survival (RFS) based on AFP expression in HCC specimens. OS and RFS were analyzed using the log-rank test. Median expression levels of AFP gene was used as the cutoff. (C) The expression of TUG1 and AFP in HCC specimens were determined by qRT-PCR. 18S rRNA was used as a loading control. Results of Pearson correlation coefficient analysis confirmed that TUG1 is significantly positively correlated with AFP.
